# Using Normalization Process Theory to Evaluate an End-of-Life Pediatric Palliative Care Web-Based Training Program for Nurses: Protocol for a Randomized Controlled Trial

**DOI:** 10.2196/23783

**Published:** 2022-11-11

**Authors:** Moustafa A Al-Shammari, Amean Yasir, Nuhad Aldoori, Hussein Mohammad

**Affiliations:** 1 Nursing Department Al-Mustaqbal University College Hillah Iraq; 2 College of Nursing University of Babylon Babil Iraq

**Keywords:** End-of-Life Nursing Education Consortium for pediatric palliative care, implementation, Iraq, life-limiting illness, pediatric palliative care, pragmatic trial, web-based training

## Abstract

**Background:**

Palliative care (PC) is a new concept in Iraq, and there is no training for health care specialists or the public. The lack of education and training programs is the most important barrier for PC. Intermediate training is needed for nurses who regularly manage patients with life-threatening diseases. The End-of-Life Nursing Education Consortium for pediatric palliative care (PPC) program is intended for nurses who are interested in providing care to children with life-limiting diseases or providing support in the event of an accident or unexpected death.

**Objective:**

Our trial aims to evaluate the effect of a web-based training course, using the Normalization Process Theory. It focuses on how complex interventions become routinely embedded in practice and on training of a sample of academic nurses in the application of PPC in routine daily practice. It hypothesizes that nurses will be able to provide PC for the pediatric population after completing the training.

**Methods:**

This is a multicenter, parallel, pragmatic trial in 5 health care settings spread across a single city in Babylon Province, Iraq. Participants will be recruited and stratified into 2 categories (critical care units and noncritical care units). In the experimental condition, 86 nurses will be trained in the application of PPC for 2 weeks through a web-based training course powered by the Relais Platform. The nurses will be invited to participate via email or instant messaging (WhatsApp, Telegram, or Viber). They will provide end-of-life care in addition to usual care to children and adolescents with life-limiting conditions. In the control condition, 86 nurses will continue usual care. The program’s effectiveness will be assessed at the level of nurses only. We will compare baseline findings (before the intervention) with postintervention findings (after completing the training course). A further assessment will be performed 3 months after the course. As numerous unidentified factors can influence the effect of the training, we will perform a progressive evaluation to assess sample selection, application, and intervention value, as well as implementation difficulties. The nursing staff will not be blinded to the intervention, but will be blinded to the results.

**Results:**

The study trial recruitment opened in July 2020. The first outcomes became available in December 2020.

**Conclusions:**

The trial attempts to clarify the delivery of PC at the end of life through the implementation of a web-based training course among Iraqi nurses in the pediatric field. The study strengths include the usual practice setting, staff training, readiness of staff to participate in the study, and random allocation to the intervention. However, participants may drop out after being transferred to another department during the study period.

**Trial Registration:**

ClinicalTrials.gov NCT04461561; https://clinicaltrials.gov/ct2/show/NCT04461561

**International Registered Report Identifier (IRRID):**

PRR1-10.2196/23783

## Introduction

### Background

Pediatric palliative care (PPC) involves comprehensive active care for children and young adults, and is a globally recognized priority in caring for those with life-limiting conditions. This recognition was justified by high rates of mortality from cancer specifically. Therefore, skilled and supportive care, pain management, and symptom control at the end of life have been approved in palliative care (PC) [1,2].

Annually, 7 million families could benefit from PPC, but those in low- and middle-income countries seldom have such access, and there are approximately 1.2 million children globally, with more males than females. These children desperately need this type of care, and they eventually die from multiple and varied problems, such as birth defects, malnutrition-related diseases, meningitis, AIDS, and cardiovascular diseases [3].

Overall, in 2011, more than 29 million individuals from diverse age groups died from diseases demanding PC, with the largest proportion being adults rather than children less than 15 years old. Globally, each year, only 63 children out of 100,000 children less than 15 years old require PC at the end of life [3].

The World Health Organization presents PC as an integrated approach involving rules, opioid availability, facility obtainability, and educational platforms, as well as PC-certified activities, and it is available in 15 Eastern Mediterranean countries. Saudi Arabia has the maximum number of PC agendas, in addition to Iran and Lebanon, who award official licensing for their physicians, followed by Egypt and Jordan. Moreover, Oman, Jordan, Egypt, and Qatar have established other advanced programs for training (eg, master’s degree or diploma). However, Iraq and occupied Palestinian areas have not started such care [4].

Some physicians and nurses have attended essential and advanced workshops on PC since 2011, but it is still considered a new concept in Iraq, with no formal policies or guidelines relating to this field, and the main contributors for introducing PC are nongovernmental organizations and the Middle East Cancer Consortium [4,5]. The number of qualified doctors is very low in Iraq, as trained physicians are fleeing the country owing to political instability. There are no degree programs in palliative medicine in Iraq, and a quarter of nurses are college graduates, with most lacking primary education [5]. No actual policy changes have been made in the previous 10 years. In the last 6 years, new opioids have been introduced, but they are not permissible for outpatients and not easily available. Only medics in government hospitals can recommend these opioids, and they are for oncology and not PC. Individual funders have started appealing to provide support for oral morphine if permitted by the Ministry of Health. There is no instant morphine or continued-release morphine. Injectable morphine, codeine, and transdermal fentanyl patches became available in 2013, which are obtained for free, and upgrading these drugs has been deliberated [4].

Obstacles to PC can be divided into the following 3 parts: (1) deficit of health policies in the sustenance of PC improvement; (2) nonexistence of significantly trained health care workers; and (3) reduced accessibility to essential PC drugs [6,7]. All these obstacles can be overcome by addressing the important barriers that are delaying PC development in Iraq, namely, lack of public responsiveness, lack of education, lack of training programs, inadequate opioid accessibility, and no identification of PC as a field [3,4].

The political state and uncertainty in the nation have played main roles in postponing the awareness of the public. Furthermore, the greatest pediatric care providers consider that PC is a good means to disclose that more cannot be done [4].

Regarding the present PC condition in many countries in the Middle East, there is a need for emphasis on the teaching of professional staff members and the presence of a consistent renewed expert committee that would be accountable for the development of the modern PC team [8-10].

PC education needs to be provided in English and is desired at the following 3 levels: (1) basic PC preparation for all health professionals; (2) intermediate training for those consistently working with patients having life-threatening illnesses; and (3) professional PC training for managing patients with more than routine symptom management needs [3].

Without appropriate end-of-life education, it is incredibly difficult for nurses to provide adequate care for related issues. Moreover, it is vital to balance education with attention to personal understanding and attitudes toward death and dying to provide students with opportunities to become knowledgeable about death and grief in order to deal with their feelings and to develop further [11]. From 1997 to 2000, there was a lack of overall nursing content related to end-of-life care and limited end-of-life content knowledge among nursing faculty [12,13]. Efforts are needed, including the development and dissemination of new educational recommendations, training materials, and educational requirements, at both the medical school and residency levels, as well as for nursing students and other health professionals, which should address the need for PC education [14].

To detect and address the limited knowledge of end-of-life care, researchers at the City of Hope National Medical Center in the United States conducted a 3-year project titled “Strengthening Nursing Education to Improve End-of-Life Care,” which was supported by The Robert Wood Johnson Foundation. The project brought together professional nursing organizations, expert clinicians, and educators in palliative/hospice care to improve the curriculum in order to enhance nursing care at the end of life. The project revealed chief insufficiencies in nursing education and its role in end-of-life care, and identified a lack of content in nursing text, insignificant content in the nursing curriculum, insufficient nursing faculty knowledge, and many other educational barriers that inhibit good nursing practice in this area [15-17].

“The End-of-Life Nursing Education Consortium-PPC (ELNEC-PPC)” project based on original training was intended to study the concern of nurses in providing care for life-threatening illnesses to children or for accidents or sudden death [18]. Perinatal and neonatal PC is one module [19]. The ELNEC-PPC train-the-trainer program is for 2.5 days, and those who complete the program will be able to actively share information and knowledge to other health care workers in clinical practice or nursing students in colleges. The first training module was started in 2003, and 8 nationwide train-the-trainer programs have been conducted with more than 700 nurses attending from varied pediatric locations across the United States and Canada.

Many children dying from life-threatening illnesses, such as congenital malformations, chromosomal abnormalities, accidents, low birth weight, and sudden infant death syndrome (50 out of every 100,000 children), could be provided PC services, but little consideration is given to reducing the suffering faced by children and their families [7,18].

Pediatric nurses are considered more occupied than any other health care professionals, and they have a distinctive opportunity to assess and address the needs of children who feel pain and die suddenly or shortly after birth, or die in utero (perinatal death), as well as the needs of their families. However, these nurses may have little knowledge about the principles of caring for terminally ill children with different conditions at different ages [20,21].

On the other hand, a problem might occur with the introduction of a new approach to supply and consolidate health care in practice, which is extensively adopted in health services, community health practice, and areas of social policy that have important health consequences, such as education, transport, and housing. The UK Medical Research Council’s framework for designing and evaluating complex interventions recommends conducting a process evaluation in order to illuminate inconsistencies between predictable and detected outcomes to understand how context impacts outcomes, and to provide insights to assist implementation [22,23].

Previous reports [24-26] mentioned that a certain problematic translational gap continues to exist between demonstrating the positive impact of a complex health care intervention in a study environment and utilizing this intervention in routine daily practice. The Normalization Process Theory (NPT) [27] and its predecessor, the Normalization Process Model [28,29], provide a framework that conceptually helps in understanding and explaining the dynamic processes that can be encountered through the utilization of complex interventions and technological or organizational innovations.

Previous studies [30,31] specified 4 key analytical domains as nonlinear and mentioned that they cooperate energetically to afford broad enlightenment of implementation processes, which anticipate if participants do not understand, sustain, or consider an intervention valuable or compatible with their current work. The NPT was designed to be applied flexibly, can be used at one or more points in a qualitative study, and has been successfully used. Following the NPT [29,31], the process of implementing a complex intervention can be described and explained by employing the following 4 central theoretical constructs:

1. Coherence: The process and work of sense-making and comprehending that individuals and organizations perform, which promote or inhibit the embedding of a routine practice.
2. Cognitive participation: The process and work that individuals and organizations perform to enroll people to build relationships in a new practice.
3. Collective action: The work that individuals and organizations perform to enact a new practice. Collective action is primarily labeled as a normalization process model and contains 4 subcomponents (“contextual integration,” “relational integration,” “interactional workability,” and “skill set workability”).
4. Reflexive monitoring: The work inherent to the formal and informal appraisal of a new practice to measure the benefits and drawbacks, which can develop users’ knowledge of the effects of a practice.

The NPT has become a widely used theory for analyzing the implementation of complex interventions and has previously been applied to a wide range of health topics and empirical settings, including chronic health care, maternity care, e-learning, and telemedicine. There have been 8 studies in the eHealth and telehealth care fields and 21 studies in several other health care fields, which entirely concluded that the NPT is a complete and valuable model to drive the process of implementation in the setting of a health facility [31].

A quantitative design in a PC health context has not yet confirmed the validity of the NPT model. The NPT tool Normalization Measure Development (NoMAD) [32] has newly been established for use in quantitative research. Similar to the Conceptual Model of Implementation Research [33], the elements are formulated to feature at application with hindsight. Besides, the customization of the NoMAD tool across several contexts is unclear, and this restricts its practical usage in selected settings. For example, a single element of the NoMAD tool tests whether “sufficient resources are obtainable to maintain the intervention” [32]. Nevertheless, to our knowledge, no NPT studies have concentrated on evaluating a web-based training program in PPC at the end of life for nursing interventions in hospital settings.

Training has been recognized as a key implementation approach for enlightening provider knowledge and skills, with an interest in the use of web-based training methods [34,35]. The indication is accumulating that web-based training can be a working educational instrument aimed at bringing and appraising curricular content through numerous organizations and training levels. With high-speed internet access and the universal presence of computers in homes and clinics, health care professionals can effortlessly access web-based resources regardless of their settings and can access resources at periods that do not overlap with their clinical tasks or responsibilities [36].

The original policy for providing consistent behavioral interventions in a series of situations involves web-based interventions, which provide the ability to access behavioral maintenance at any time, and result in greater confidence and lower cost compared with clinician-delivered interventions and old-fashioned face-to-face training methods [35,37].

Survey and qualitative studies mentioned that end-of-life care research is feasible and ethical, but funding of end-of-life care research is poor [38,39]. Thus, randomized trials of end-of-life care treatments and services are uncommon, and are often limited by poor enrollment, high attrition, unfairness, confusion, and small samples [40,41].

Specifically, pragmatic multicenter randomized controlled trials (RCTs) are recommended for the evaluation of complex health care interventions to enrich enrollment and broadcasting, and increase the external validity of trial results [42]. Nevertheless, pragmatic RCTs are intended to evaluate treatments in real-world (as opposed to idealistic) conditions, directly enlightening decision-making by patients, providers, and health care policymakers [43].

### Comparators

Comparators are often used to decide the work level of an intervention related to a clinically relevant substitute. The choice of the comparator (control group) is always a serious decision in a clinical trial, which has the following major purpose: to allow the differentiation of patient outcomes (for example, changes in symptoms, signs, or other morbidities) triggered by the test treatment from outcomes caused by other factors, such as the natural advancement of the disease, observer or patient potentials, or other treatments. The comparator experience helps identify outcomes when patients do not use the test treatment or when they use a different treatment [44].

The present knowledge regarding the absolute value of anticipated experimental and control interventions is considered as one of the factors that regulate the appropriate choice of a comparator for a trial [45]. The research question type is the main ideal comparator determining factor. Significant comparators are those that involve existing clinical or public health services or facilities (eg, usual care or standard care), other implementations (eg, a firm evidence-based practice intervention by way of a comparator aimed at an intervention that is up to date), and clinically pertinent variations in trial implementation (for instance, similar interventions employing an alternate approach, eg, a face-to-face intervention in studies assessing a telehealth intervention) [46].

The pragmatic model intended for the selection of comparators allows investigators to choose clinically important comparators instead of nonnatural comparators that are unlikely to continually be used in clinical practice. The pragmatic model provides a way to resolve the numerous incongruities and influences related to comparators that are used in intervention trials [46].

For this trial, typical care is the comparator of choice, which is considered as a treatment or service that is consistently used, and it is provided after experimental members have joined. It often differs across individuals and settings, and in some trials, it may be increased or decreased for trial participants [46]. According to a previous report [47], customary care is the favorite comparator among researchers. It has been substituted as a comparator or has been a constituent of the comparator in 99 (49.5%) studies, and often appears under numerous synonymous terms (eg, standard treatment, care, as usual, standard care, treatment as usual, and standard of care). However, a previous report [48] stated that it may vary considerably between centers and countries confounding comparator choice. Using clinical guidelines to define usual care can help standardize comparator treatments; conversely, this may decrease the applicability of the results to actual settings.

Hereafter, the selection of typical care for this trial follows Clinical Practice Guideline recommendations, as well as the organized appraisal of significant literature that appears to be related to PC services, which are not accessible to pediatric patients in Iraq owing to deficiency of knowledge and training among health care professionals or limited care supply [4,10,45]. The trial aims to explain the provision of PC at the end of life through the implementation of the ELNEC course as a web-based training program, using the NPT. Its emphasis is on the complexity of interventions, which are routinely embedded in practice. In addition, the trial aims to identify the changes implemented by participant nurses in their clinical practice after participating in the web-based training program to provide PC alongside usual care (intervention group) versus usual care only (control group) for children with life-limiting conditions or in the case of accidents or sudden death at the end of life. Finally, we want to provide findings that will assist in the interpretation of the trial results.

### Objectives

#### Primary Objective

The primary objectives are as follows: (1) to hold a short ELNEC-PPC web-based training program at selected Hillah city hospitals in Iraq by July 2020, and (2) to evaluate the impact and effectiveness of this project through the NPT at the beginning of the web-based training course, and 2 weeks and 3 months after the end of the web-based training course, define PC advocacy activities, and implement the principles found in the ELNEC-PPC web-based training program at selected Hillah city hospitals in Iraq by August 2020.

#### Secondary Objective

The secondary objective is to monitor participants for 3 months after the web-based training program in an attempt to raise their PPC knowledge to improve their self-efficacy levels and attitudes toward PPC at selected Hillah city hospitals in Iraq by August 2020.

## Methods

### Trial Design

This study is designed as a multicenter, investigator-blinded, parallel, 3-month, pragmatic, 2-arm, superiority (provision of PPC with usual care is superior to usual care only) RCT, in which different units from multiple hospitals are stratified and randomized. Randomization will be performed as block randomization with 1:1 allocation, and it will be conducted according to the Consolidated Standards of Reporting Trials (CONSORT) guidelines. The CONSORT statement is a guideline designed to improve the transparency and quality of the reporting of randomized trials [49,50].

### Study Setting

The trial will take place in selected Hillah city hospitals, which are local government organizations that have various pediatric and adult sections. In Iraq, there are 273 public hospitals spread all over the country [51]. Hillah city in Babylon province has 4 hospitals and 1 Babylon Oncology Center. The recruitment of participants will be conducted at the following 5 locations: Imam Sadiq Teaching Hospital (general hospital) with 492 beds, Babylon Maternity and Children Teaching Hospital (obstetric and pediatrics hospital) with 323 beds, Morgan Teaching Hospital (specialized center for tertiary health care), Al-Noor Hospital for Children (pediatrics hospital), and Babylon Oncology Center [52].

Participants will be recruited and stratified into the following groups: (1) critical care units (1 artificial kidney unit, 1 resuscitation emergency unit, 1 catheterization unit, 2 children’s emergency units, 1 emergency department, 1 maternity emergency unit, 1 morning resuscitation unit, 1 operation room, and 1 pediatric surgery unit); and (2) noncritical care units (1 blood disease unit, 1 chemo injection unit, 1 health insulation unit, 1 private suite, and 5 pediatric lobby units). Staff in the selected hospitals are not employed in 2 areas at the same time, and pediatric patients with several diagnostic classifications are managed and provided usual care. Recruitment for this study started in July 2020. There is a 2-year outline plan for the research, with likely changes along with primary analysis outcomes.

### Inclusion and Exclusion Criteria

The study will include nurses who (1) have been employed by Imam Sadiq Teaching Hospital, Babylon Maternity and Children Teaching Hospital, Al-Noor Hospital for Children, Morgan Teaching Hospital, and Babylon Oncology Center; (2) have completed their bachelor’s degree and have a master’s or doctorate degree in nursing sciences; (3) have been working for at least 3 months and are not expected to be moved to another internal unit during the research period (including morning and evening shifts); (4) provide nursing care for both male and female hospitalized patients aged ≤18 years; (5) use a computer (desktop or laptop) with access to the internet at home or work (phone line or internet access), or use a smartphone (Android 6.0+ or iOS 11.0+) with internet access (Wi-Fi or mobile data) to join the online training course; and (6) have a working email address or a working mobile number, and have access to a computer or smartphone with internet access to complete questionnaires in a web browser.

The study will exclude nurses who (1) are not interested; (2) are not employed for at least 3 months; (3) work in units other than the selected units; and (4) are enrolled in another experimental trial.

The evaluation of whether the exclusion criteria are met is performed by the investigator during the recruitment phase or according to the participant’s responses during the interview.

### Screening for Eligibility and Enrollment

The recruitment period is planned to start in July 2020. A total of 172 participants will be recruited in the RCT. Of these, 46.5% (n=80) will be recruited from Imam Sadiq Teaching Hospital, 26.2% (n=45) will be recruited from Babylon Maternity and Children Teaching Hospital, 7.6% (n=13) will be recruited from Morgan Teaching Hospital, 16.3% (n=28) will be recruited from Al-Noor Hospital for Children, and 3.5% (n=6) will be recruited from Babylon Oncology Center. Collaborations will be established with the selected hospitals to facilitate recruitment. The number of participants needed to ensure a sufficient recruitment rate has been determined (see the sample size section).

**Figure 1:**
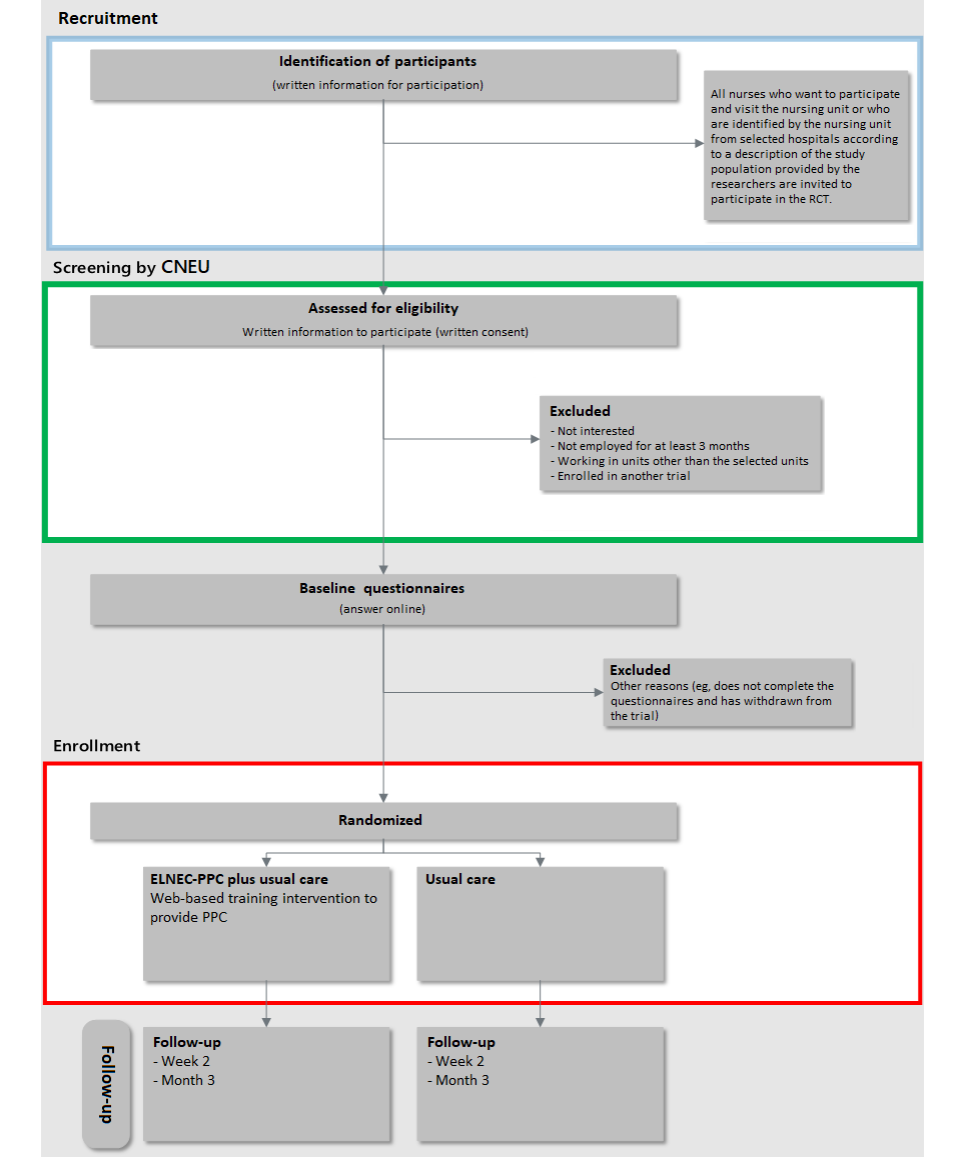
Participant flow through the trial. CNEU: Continuing Nursing Education Unit; ELNEC: End-of-Life Nursing Education Consortium; PPC: pediatric palliative care; RCT: randomized controlled trial.

The recruitment process is outlined in Figure 1. The main investigator will submit the official approval form for conducting the research at the selected hospitals (Multimedia Appendix 1), and the approval form for the training course (Multimedia Appendix 2 and Multimedia Appendix 3), which will be hosted by the Continuing Nursing Education Unit (CNEU) at the Faculty of Nursing.

Eligible nurses will be invited to participate in the trial by the CNEU team in the selected settings. The unit will refer potentially eligible participants depending on a quick eligibility explanation that the main investigator submits to the unit. The documents are as follows:

1. Nomination form for the training course (Multimedia Appendix 4) for recruitment: Each participant will fill out this form, and the data will be used for contact with each participant. The data fields include name, email address, telephone number (Telegram, Viber, or WhatsApp service), current duty, type of practice unit inside the selected hospital, date, job description, academic qualification, a text box for random numbers in the upper left side of the form, and participant’s signature. The number of forms printed is according to the size of the estimated sample for the research (see the sample size section).
2. Table of the estimated sample: This table presents the details of the estimated sample and the types of practice units inside the selected hospitals (Multimedia Appendix 5). Using this table, the person in charge of the CNEU in each selected hospital will select eligible participants.
3. Informed consent form (Multimedia Appendix 6)

Referral to the trial will take place after the CNEU in each selected hospital has selected participants according to the table of estimated sample size that will be provided to the CNEU and the current practice unit (usual procedure). Participants will be recruited through the unit team in the selected hospitals. Participants are provided with an invitation letter that includes written information about the project and numbered, opaque, sealed, and stapled envelopes that contain the nomination form for the training course. The written information provides 2 ways to contact the researcher, if the participant is interested in the project: (1) by email and (2) by telephone (call or instant message by Telegram, Viber, or WhatsApp message). The nomination forms for the training course filled out by candidates are placed in large (A4) sealed envelopes and sent to the CNEU at the Faculty of Nursing that will host the web-based training course.

The start of the study will involve preparation of formal and essential approvals through the academic professor (HJ) responsible for the CNEU at the Faculty of Nursing and coordination with the Babylon Health Department, to obtain official agreements for sample collection according to the study criteria. The candidate envelopes will be opened and the candidates will be entered in an Excel spreadsheet. The information entered in each form will be added in sequence, and the Excel spreadsheet will be backed up, printed, and kept in a safe and secure location. A copy of the spreadsheet will be sent via email to the main investigator to contact the participants via their provided email addresses or phone numbers (Telegram, Viber, or WhatsApp). The enrollment process is outlined in Figure 1. If candidates are eligible and willing to participate, the informed consent form will be signed and the main investigator will contact each participant and send a link to the web-based baseline questionnaire via email or social communication apps (Telegram, Viber, or WhatsApp). Then, randomization will be performed via a web-based randomization system (see the randomization section).

Participants can return the signed informed consent form in a prestamped envelope provided by the unit or can take a picture of the signed consent form and send it via a text message to the main investigator. Subsequently, the main investigator will contact the participants, inform them about the results of the random distribution, and provide instructions according to the group that was randomly chosen (control or intervention group). All instructions will be completed on the phone or via text message, and participants will receive a link to a website from the main investigator (Multimedia Appendix 7), which provides all instructions and details of participation in the training course (also presented in Arabic).

In the CNEU, randomization will be performed. If randomized to the ELNEC-PPC web-based training course in addition to usual care (intervention group), participants will be sent a link to the website that has all instructions for enrolling and registering in the training course and assistance on how to maintain access via instant messaging or email. Moreover, participants will be given information about their follow-up assessments and how to contact the main investigator if needed. If a participant declines to participate, the main investigator will ensure that the information from the baseline questionnaire is deleted. If randomized to usual care, participants will be instructed on the principles of usual care and will be given the same information as provided to the intervention group about their follow-up assessments and how to contact the main investigator if needed. The flow of participants through the trial is described in Figure 1.

### Randomization

Participants will be randomized to either (1) ELNEC-PPC web-based training as well as usual care or (2) only usual care. The randomization involves block randomization plus permuted blocks of random size known to the researcher and stratified by the type of unit (ie, critical care unit and noncritical care unit). The allocation ratio between the intervention and control groups is 1:1. Randomization will be performed using the Sealed Envelope website [53].

Randomization involves generating random numbers using a computer. The numbers are distributed and written on the nomination forms for the training course in the text boxes on the top of the forms. According to the sample size, the first random number represents the intervention group and will be written on the form with the code “T,” and the random number for the control group will be written with the code “C.” The forms are placed in sealed envelopes, and these are mixed and placed in a large sealed envelope (A3) (Multimedia Appendix 8). These steps represent the randomization process performed by a nurse. A video is made of the process of preparing the sealed envelopes, with the participant details visible. A second researcher will later view the video to ensure the accuracy of the process. Corresponding envelopes will be opened only by the participants who select them, and they will be allocated accordingly. The enrolled participants will complete all baseline assessments.

### Blinding

This is a single-blinded study, and the participants are not blinded to group allocation. Examination and interpretation of the study results will be achieved by investigators blinded to group allocation. Once the study is completed, a copy of the data will be extracted in a pseudonymized form for statistical analyses. The information concerning the group allocation will be added to the data set with the intervention and control groups categorized as T and C, respectively. This work is done under a camera for documentation later (by supervisors), and the complete data entry is performed with blinding of the main researchers. The randomization key (ie, document with group details) will be kept with supervisors. They will provide the randomization key to the researchers once blinded interpretation of the results is finalized.

### Interventions

Labeling will be performed according to the SPIRIT (Standard Protocol Items: Recommendations for Interventional Trials) recommendations [54] and CONSORT EHEALTH extension [55].

#### Usual Care

Participant nurses deliver usual care according to their roles appropriate for neonates, infants, toddlers, preschoolers, school-age children, and adolescents in the selected units of perinatal, neonatal, and pediatric settings. It comprises all treatment management procedures, diagnostic procedures, and referral processes, which are considered relevant in terms of case history, clinical results, and practical everyday practice. After trial completion, participants in this group will be allowed to access a web-based training course similar to that provided in the intervention group.

#### PPC With Usual Care

The online ELNEC-PPC course is being developed in collaboration with Relias Learning Systems [56]. The ELNEC-PPC course involves complete nationwide work to advance palliative end-of-life care delivered through health care experts in perinatal, newborn, and pediatric contexts. The mission depends on the unique ELNEC care course training, which involves cooperation between “The City of Hope” and the American Association of Colleges of Nursing. This design may include a combination of investigation and knowledge in PC and is meant to help with applying evidence-based practice. The American Association of Schools of Nursing Peaceful Death: Recommended Competencies and Curricular Guidelines for End-of-Life PC and the 1997 Drug Report have been considered. The electronic training course is going to be included in modules that are for 2.5 days, and those who complete the ELNEC-PPC course will be able to take the knowledge to clinical practice [18]. The curriculum involves the following 9 modules that are specific to the care of children who have life-limiting illnesses, and their families [57]: module 1, nursing PPC introduction; module 2, perinatal and neonatal PC; module 3, PPC communication; module 4, PPC ethical/legal issues; module 5, PPC cultural and spiritual considerations; module 6, PPC pain management; module 7, management of symptoms; module 8, PPC loss, grief, and bereavement; and module 9, PPC at the time of death.

Each module will cover about 20 minutes of PowerPoint slides and data text, and 40 minutes of activity sessions for clinical application. The clinical course will comprise case management studies, film vignettes including critical thinking questions, and “stop and think” queries that require the user to answer in order to continue through the module. On the completion of each training module, the participant will be requested to reply to 10 National Council Licensure Examination–format questions to complete mastery. This interactive online course is important for all participants in the selected Hillah city hospitals.

The following 8 main themes are present within each of the modules: (1) Family as a unit of care; (2) Important role of the nurse as an advocate; (3) Cultural importance as an influence in PC; (4) Critical demand for notice to special populations like ethnic subgroups, deprived individuals, and uninsured individuals; (5) PC influence on care systems according to context; (6) Critical monetary matters impact PC; (7) PC is not limited to cancer or AIDS, and is vital in serious diseases and cases that can result in sudden death; and (8) Interdisciplinary care is vital for end-of-life care [18].

It has been hypothesized that the NPT would be a useful conceptual tool because it provides a strong analytic framework for understanding the organization and operationalization of tasks (their implementation), creating them as routine elements of lifestyle (their embedding), and sustaining embedded practices in social contexts (their integration) [28].

### Process of Implementation

Institutional review board approval will be obtained from Babylon University/Nursing Faculty and from the selected hospitals in Babil Province Health Department, where the study will take place. Informed consent will be submitted to the selected hospitals, and the nurses will be given 1 week to decide if they wish to participate. The Arabic NoMAD pretest electronic questionnaire will be provided to those nurses who decide to participate.

The intervention group will receive the ELNEC-PPC training course through the Relais Academy website. The main investigator will send a link of the e-questionnaire to all participants via their email addresses or via social media (Telegram, Viber, or WhatsApp). It includes questions that describe the experience and role of each participant in providing PPC, and within 2 weeks, each participant will return to their appropriate routine practice. The time periods from pretest (the ELNEC-PPC training intervention) to posttest will be 2 weeks and 3 months.

The main investigator will send a link of the website that provides details of participation in the training course and enrolling at Relias Academy (see link in Textbox 1). The website also provides support in Arabic to all participants in the intervention group. For enrolling at Relias Academy, the participants need to follow the process presented in Textbox 1.

**Process for enrolling at Relias Academy.**

1. Go to www.reliasacademy.com
2. Select “Sign In” from the top right corner of the screen
3. Type in your email address and password
4. Email: Provide the email
5. Password: Provide the password
6. Confirm that the End-of-Life Nursing Education Consortium-Pediatric Palliative Care course you are looking for is present
7. Once logged in, click on “Manage Account” and select “Courses”

Participants will login to the site from the link sent to them, and will read the steps necessary to complete the course (our website will provide the information). The site was designed for research purposes by the researchers to deliver the content of the course that was on the Relais Academy platform and provide the required survey to the participants to facilitate the process of implementation. It was created using Google Site, a service provided by Google to build websites, and the way it works is similar to the way a wiki works.

Each participant in the intervention group will complete all 9 modules of the ELNEC-PPC training course. Meanwhile, the main investigator will follow-up with the participants entering the website link and ask them via an instant message or email about the completion of steps and upload of a screenshot for each module to a Google Form URL provide to them.

After 2 weeks, the main investigator will make sure that all participants in the intervention group have completed the tasks assigned to them during the course, by asking the participants via an instant message or email, and will also send certificates for each module. Then, the electronic questionnaire (Arabic NoMAD) will be sent again to be filled out for the second time by the intervention group, and the main investigator will make sure that all the forms have been completed within 2 weeks after the training course. After that, the participants will be monitored at their workplaces for 3 months in order to measure the impact of the training course on their work and the changes that affect their roles, as well as evaluate their experiences using the NPT toolkit. Interviews will be conducted at their workplaces in the selected hospitals.

After 3 months, the main investigator will measure the variables of the interview using the NPT toolkit to create a viewpoint for PPC delivery via nurses at the end of life, and assess the modality of working in the hospital units. Simultaneously, a NoMAD link will be sent to all participants from the 2 arms for the last time to evaluate the long-term impact of the training course on providing PC.

### Outcomes

All outcomes of the study will be assessed at the starting point, and after 2 weeks and 3 months. Participant characteristics and demographic variables will be collected at baseline. Participant characteristics include age, gender, and relevant comorbidities, while demographic variables include job category, work experience, academic qualification, job title, and the number of local training courses. The outcomes are based on the recommendations of the NPT and its 4 related constructs [58].

#### Primary Outcome

The primary outcome involves the evaluation of the ELNEC-PPC course aimed at providing PC in a range of health care contexts through the Arabic NoMAD questionnaire and observation of routine clinical care.

#### Secondary Outcome

The secondary outcomes involve an interview, operating context examination, and assessment via the NPT toolkit.

### Interview Procedure

Semistructured face-to-face interviews will be conducted with all nurses on successfully completing the ELNEC-PPC web-based training course. All participants have direct contact with patients, and 3 rounds of interviews will be conducted after 3 months. After consenting, participants will be interviewed by the main investigator (MA). All interviews will be audio recorded and transcribed. If participants do not want to be recorded, the main investigator will make notes and then transcribe an in-depth discussion account. Some participants will be interviewed in pairs or small groups. They will be asked about the changes they experienced in terms of care after the training. Throughout the interview rounds, participants will be questioned about the most important developments since the start of the program. They will be asked to disclose their opinions around PPC and to clarify the degree to which they would be implementing the method. Themes are based on the NPT [59] and will be reviewed to include problems from the initial meetings. This will assist researchers in exploring participant views on the simplest models of care.

Consequently, the interactive NPT toolkit will be used. It contains 16 questions for thinking through an implementation problem. The embedding will be improved, and statements and explanations will be edited for a web-enabled tool.

### Sample Size

The study has been designed as a superiority trial with 2 parallel groups (ELNEC-PPC web-based training plus usual care and usual care only). For calculation of the appropriate sample size, the main researcher uses the assumption that the 5 selected settings have 254 nursing staff members on average eligible for inclusion based on a survey of all included settings before the study to determine the total number of nurses who work in the selected settings and are appropriate for inclusion. Based on this assumption, as well as a 2-sided *P* value <.05, a 0.90 power, a traditional predictable correlation between 2 measurements of 0.5, and an intraclass correlation coefficient with *P*<.05, 86 participants were required in the intervention group and 86 were required in the control group for *t* tests. To support equal sample sizes, including for the units of the selected settings, and to account for intrastratified correlations of units, a multilevel analysis will be used. Nursing staff members who are transferred to a dissimilar organization unit will be exchanged from the sample of nursing staff, and changes will take place in the course of the study. To catch up with nursing staff members who are not changed in time, the researchers will insert an additional organization unit from the critical care units for the intervention and control groups, which will result in a total of 172 nursing staff members. All power analyses will be performed using the G*Power software (version 3.1).

### Data Collection

#### NoMAD Instrument

Participants will complete 2 data collection forms at the beginning, and forms will also be completed after the ELNEC-PPC training. The main data collection instrument is NoMAD [33]. The original form of this instrument has been translated into Arabic and adapted to Arabic conditions to evaluate the normalization potentiality of PPC training, which will be provided by a web-based training intervention.

The NoMAD instrument has been translated into the Fusha dialect in translation steps outlined previously [60]. The authors translated the NoMAD instrument from English to Arabic, and then, back translated it to English. The content validity and translated NoMAD acceptability have been measured in a reiterative procedure involving several recognized steps, including translation forward and translation backward, tests of the target language instrument content validity, meetings with specialists, and further revision, as well as a final content validity test of the studied tool. The finalized tool has been created as an electronic form. The Arabic NoMAD prime version, following step 2 of the clarification and adaptation process, has been applied in a pilot study. The Arabic NoMAD instrument is split into the following 3 sections: Section A, which includes 12 items on the respondents; Section B, which includes 3 general items on the intervention; and Section C, which includes 20 identifiable items on the intervention. Regarding the 4 NPT concepts, “Coherence” has 4 questions, “Cognitive Participation” has 4 questions, “Collective Action” has 7 questions, and “Reflexive Monitoring” has 5 questions.

The Arabic NoMAD scale consists of 31 Likert-type items. The items in section B are rated on a 10-point Likert-type scale from “not at all” to “completely.” The items in section C are rated on a 5-point Likert-type scale from “disagree strongly” to “agree strongly.” “Neutral” and “not applicable” are also assumed as choices to explain respondents’ experiences of using the intervention within the workplace (Multimedia Appendix 9). A total of 30 participants have completed the Arabic NoMAD survey, with a 100% reply rate. Content validity computed for the Arabic NoMAD scale (content validity index [CVI]) was 0.91, which is significantly higher than the suggested level of 0.80, and the item-CVI was 0.71-1.00. Scale reliability was also strong, with a Cronbach α coefficient of .77-.86 in the postcourse analysis. In a recent study, the NoMAD instrument demonstrated an indoor consistency score (α coefficient) of .76-.83 [61], and therefore, it may be considered as reliable.

The NoMAD questionnaire has been translated into 7 languages [62]. The Arabic NoMAD instrument provides a versatile “bank of items” [63], with a focus on wide item versions, for instance, to supply more anticipatory assessments. The NoMAD designers propose that the tool must be examined as a “pragmatic measure” of an intervention [64,65], and it motivates adaptation for multipurpose functions in particular application studies and meets clinical practice requirements.

The Arabic NoMAD instrument has 4 building item groups, according to reliability and validity data. In addition, there is no proposal for definite scoring instructions or construct procedures, which should be used in each study.

The time to complete the baseline questionnaire is approximately 20-25 minutes. The follow-up questionnaires are shorter, and the time to complete these is approximately 20 minutes.

At the starting point and 2-week and 3-month follow-ups, participants will be sent an email with a link that directs them to complete the Arabic NoMAD questionnaire. To ensure as high a response rate as possible in the follow-up questionnaires, several reminder emails will be sent (every 3 days). If no response is received, the investigator will attempt to communicate with the participant via text message or phone call and inquire if the participant is ready to reply to the survey over the telephone.

#### Interviews

Semistructured face-to-face interviews will be conducted with all nurse participants to detect the level of successfully passing the ELNEC-PPC web-based training course from the selected settings. All participants have direct contact with patients, and 3 rounds of interviews will be conducted after 3 months. After consenting, participants will be interviewed by the main researcher (MA). All interviews will be audio recorded and transcribed. If participants do not want to be recorded, the researcher will take notes and subsequently write a detailed account of the discussion. Some participants will be interviewed in pairs or small groups. They will be asked about the changes they experienced with regard to training.

The interactive NPT toolkit will be used for assessment. It contains 16 questions for thinking through an implementation problem. The embedding will be improved, and statements and explanations will be edited for a web-enabled tool.

### Access to Data

All personally identifiable data collected in the trial will be kept for 5 years. These data are kept to be able to track any adverse events reported after completion of the trial. After these 5 years, the data set will be fully anonymized. The anonymized full data set will be kept for up to 30 years for research purposes and will be used to create a data model that can inform the further development of potential research of ELNEC-PPC web-based training. Data will be stored at the Postgraduate Department of Nursing College/Babylon University.

### Statistical Analysis

Results will be stated according to the CONSORT declaration concerning health [49,50]. The analysis of primary data will follow the intention-to-treat principle and relate the alterations in the overhead outcome measures between the intervention and control groups. SPSS Version 26.0 software (IBM Corp) will be used for the data analysis. Descriptive statistics will be presented by percentage distributions and indicators describing the location (arithmetic mean=M) and deviation (range; standard deviation=SD). The analysis of primary data will predict the mean difference with 95% CI in the NoMAD score at the 3-month follow-up between the intervention and control groups.

The model includes accessible data of nurse participants at all time points (ie, starting point, and after 2 weeks and 3 months). Within the regression model, distinct participants will be considered using a random effect approach, accounting for inside topic covariance construction. The group and time effects will be considered with asset effects using a combined intervention and time variable. Starting point levels will be obtained over 2 research collections supposing that starting point alterations are coincidental [66]. This is aimed at minimal starting point changes in the outcome variable.

Both groups will primarily be labeled according to their starting point features. The initial data will be analyzed to evaluate the effectiveness of ELNEC-PPC web-based training in the intervention group when compared with the findings in the control group, according to the NPT.

The analysis will inspect intervention effects continuously over time, and will assess the interaction between period and group allocation. The difference between the groups will be assessed for the period of the basic model and will be further evaluated according to the stratification by unit category (critical care unit and noncritical care unit) [67].

Repeated measures ANOVA will be performed to assess the multivariate main intervention effects (associated with controls) considering pre, post, and follow-up time points, as well as their interface effects. A 2-sided *P* value <.05 will be considered significant. The standardized effect size (Cohen *d*) will also be calculated. Mediator analysis will be controlled to determine which subgroups would benefit further from the intervention, with outcome variables regressing on independent variables, including age group, sex, education, and starting Arabic NoMAD score.

Secondary outcomes will be analyzed using an approach similar to the approach described for the primary outcome with linear mixed models for repeated measures. The data from the 2-week and 3-month follow-ups will also be analyzed using the approach described above for the primary outcome.

### Pilot Testing

A pilot study was conducted between March 2020 and July 2020, by using the initial Arabic NoMAD version, following clarification and adaptation. This pilot study was the initial implementation of the PPC web-based training intervention.

The web-based training intervention was used for the provision of PPC in hospitals by staff nurses, and for the application and development of further adaptable and improved operating approaches in health care facilities.

The pilot study was performed to test the instruments, make revisions where necessary, and again test the instruments. Other aspects of the research, such as how to gain access to respondents, were also piloted. Moreover, we tested a process to implement the ELNEC-PPC web-based training intervention, and to gain information about practical procedures regarding recruitment and screening as described in this protocol. Accordingly, the pilot study identified challenges in the recruitment process that could be addressed before the RCT.

The pilot study was conducted with the methods described for the RCT in this protocol. Recruitment ran until testing from all described channels. All participants in the pilot study contributed to the ELNEC-PPC web-based training and usual care (intervention). Outcome data were collected at baseline and after 2 weeks. The outcome data collected will not be included in the RCT analysis.

### Ethics Approval

Approvals for the pilot study, RCT, and process evaluation have been obtained from the relevant ethics committees in Nursing College/Babylon University. Approval was sought from the Committee on Scientific Research Ethics (number 291; January 29, 2020) and Babel Health Director (number 124; January 30, 2020). Correspondingly, approval from institutional review boards for the protection of data activities has been obtained within Nursing College. The trial has been registered at ClinicalTrials.gov (NCT04461561).

For this trial, serious adverse events are not expected, and thus, no interim analysis or a priori stopping rules are defined or implemented for this trial. All inquiries from participants reporting technical or medical problems will be registered. The website for providing the training course contains a link to a webpage with frequently asked questions that can guide participants with technical issues. All inquiries will be documented and conferred in an internal review, and the research outcomes will be described.

## Results

The RCT results will be presented in compliance with the CONSORT 2010 writing recommendations, in addition to the 2013 modification (CONSORT-EHEALTH) focused on writing mobile-based and web-based RCTs [50,55]. Data collection is expected to be completed by March 2021, and the distribution of trial results is planned after analysis.

## Discussion

**Figure 2:**
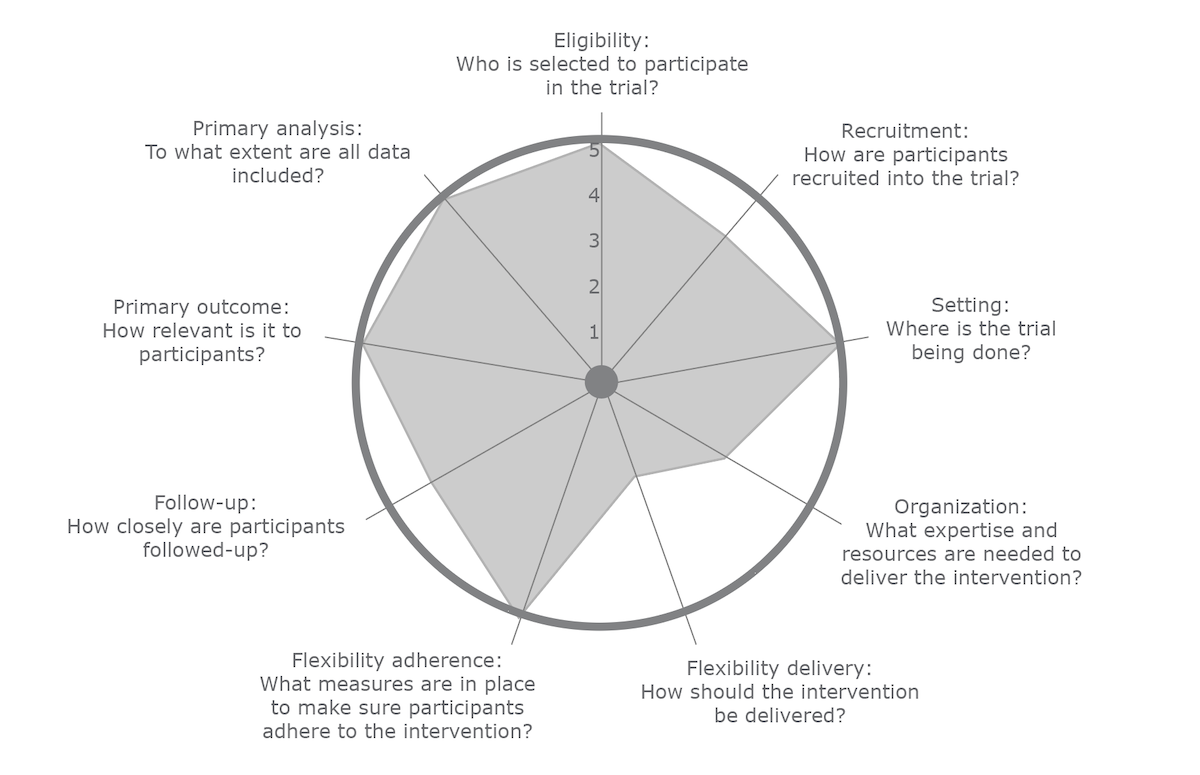
Illustration of the End-of-Life Nursing Education Consortium-pediatric palliative care web-based training intervention trial according to the Pragmatic Explanatory Continuum Indicator Summary-2 wheel.

To the best of our knowledge, this RCT will be the first trial to clarify the delivery of PC at the end of life through the implementation of ELNEC-PPC as a web-based training course among Iraqi nurses in the pediatric field. In addition, it will be one of the limited trials to evaluate enhancement in PC as a result of the ELNEC-PPC web-based training program. It will also evaluate the impact and effectiveness of this program by using the NPT, which focuses on how complex interventions become routinely embedded in practice. The study design has several strengths, including random allocation to dissemination conditions, the inclusion of data on reach, implementation under different conditions, the willingness of staff to participate, and the highly pragmatic nature of the protocol, with planning via the Pragmatic Explanatory Continuum Indicator Summary (PRECIS)-2 guidelines [68] (Figure 2) that show the characteristics of the ELNEC-PPC web-based intervention. The mean score among the 9 domains of the PRECIS is 4.22, which indicates a pragmatic protocol, with training of staff members who work in pediatric units and mixed units under the experimental condition. An important strength of the intervention is its modification according to the requirements and wishes of the health care staff to overcome hindrances, which will benefit health care administration. To increase nursing staff motivation, the web-based course will be provided to participants in the control group on completion of the study.

The trial has some limitations that must be mentioned. First, the participants will be aware of the intervention being received, which might cause bias. Second, as self-reported data will primarily be used, there might be recall bias. Third, a high dropout rate is likely, even with self-choice and additional strategies to reduce it (for instance, email notices in addition to further incentives). Fourth, the outcomes will be assessed up to only 3 months. Finally, the self-administered web-based intervention might be less efficient.

Up to now, the readiness of nursing staff to join the ELNEC-PPC web-based training course has been outstanding. The first training was provided in March 2020 for the pilot study, and the last training was provided in August 2020. The results became accessible in December 2020. The study will involve numerous departments. All units have a bottom-up approach for offering PC to children with life-limiting illnesses.

The selected design is appropriate for the study purposes. The design permits evaluating the training program’s influences on normalization and adaptation for providing PPC during daily routine practice promptly. In addition, a brief assessment will be performed at 3 months.

We hope that the findings of this study show that the web-based training intervention not only supports nursing staff in providing PPC in addition to usual care, but also has a positive impact on high-quality nursing care in pediatric governmental health care organizations and provides knowledge about recovery in children with life-limiting illnesses. Health care workers will be able to provide original information intended for modifying the intervention according to their patients’ needs. The results will help health care policy makers decide whether to expand the ELNEC-PPC program to all nursing staff in the country.

## Appendix

Multimedia Appendix 1 Official approval form for conducting research.

Multimedia Appendix 2 Approval form for the training course.

Multimedia Appendix 3 Additional approval form for the training course.

Multimedia Appendix 4 Nomination form for the training course.

Multimedia Appendix 5 Details of the estimated sample needed.

Multimedia Appendix 6 Informed consent form.

Multimedia Appendix 7 Full website screen (in English).

Multimedia Appendix 8 Randomization process by a nurse.

Multimedia Appendix 9 Normalization Measure Development questionnaire.

## Abbreviations

CNEU: Continuing Nursing Education Unit
CONSORT: Consolidated Standards of Reporting Trials
CVI: content validity index
ELNEC: End-of-Life Nursing Education Consortium
NoMAD: Normalization Measure Development
NPT: Normalization Process Theory
PC: palliative care
PPC: pediatric palliative care
PRECIS: Pragmatic Explanatory Continuum Indicator Summary
RCT: randomized controlled trial
