# Supplementary figures and images for: Using Normalization Process Theory to Evaluate an End-of-Life Pediatric Palliative Care Web-Based Training Program for Nurses: Protocol for a Randomized Controlled Trial
Source: JMIR Res Protoc. 2022 Nov 11;11(11):e23783. doi: 10.2196/23783 (PMC9700242; doi:10.2196/23783)

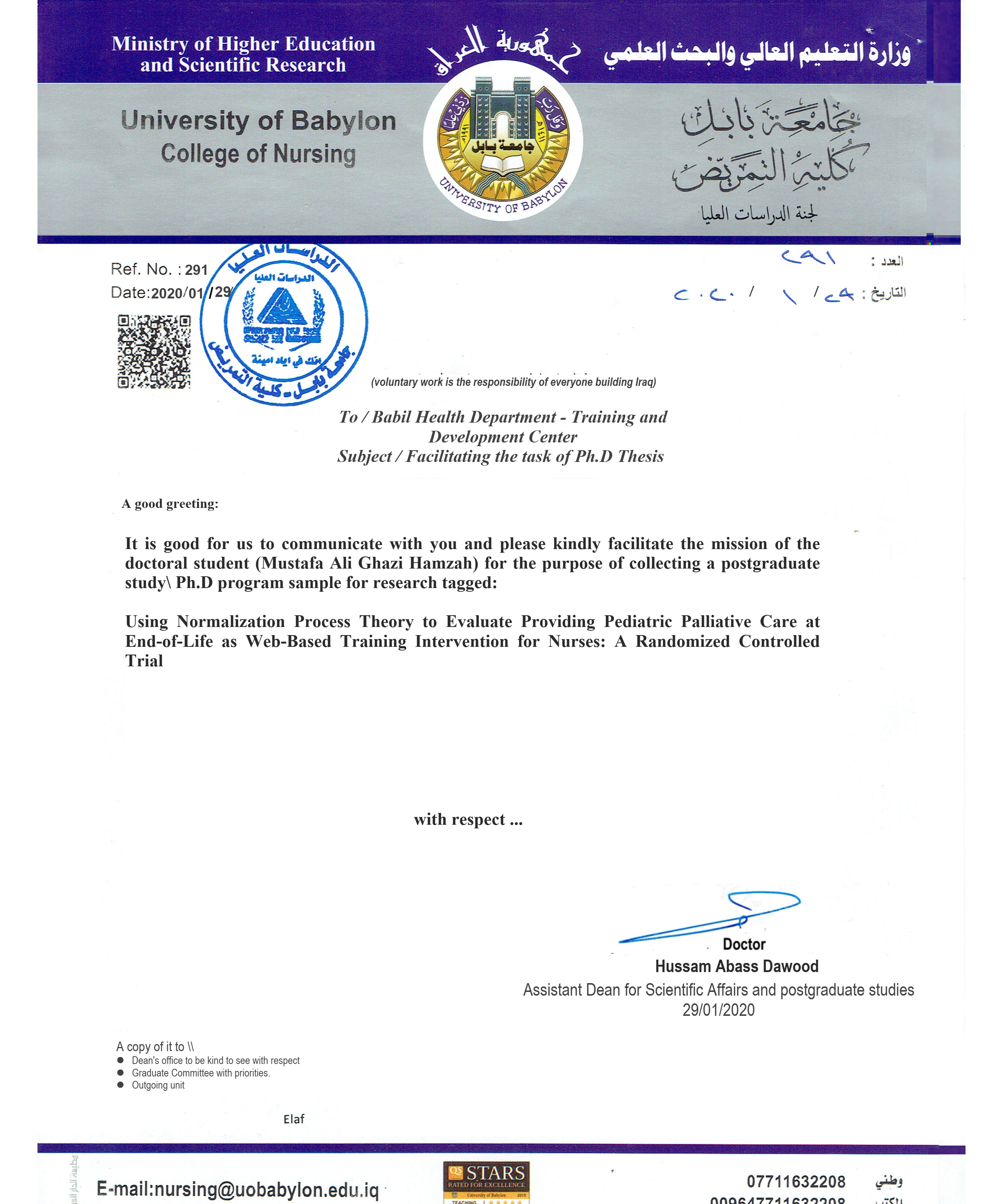

Supplement: Multimedia Appendix 1 [file resprot_v11i11e23783_app1.png]

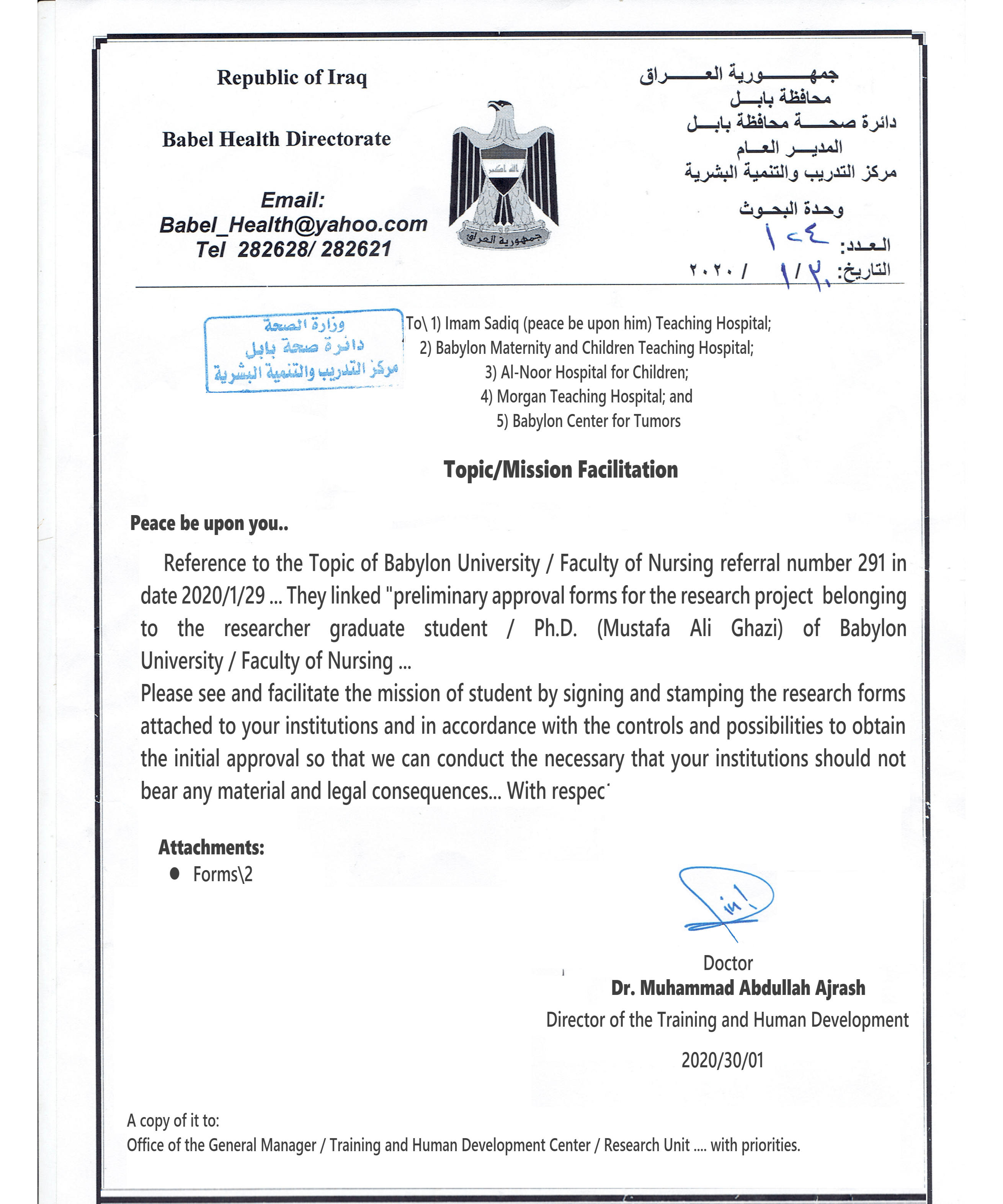

Supplement: Multimedia Appendix 2 [file resprot_v11i11e23783_app2.png]

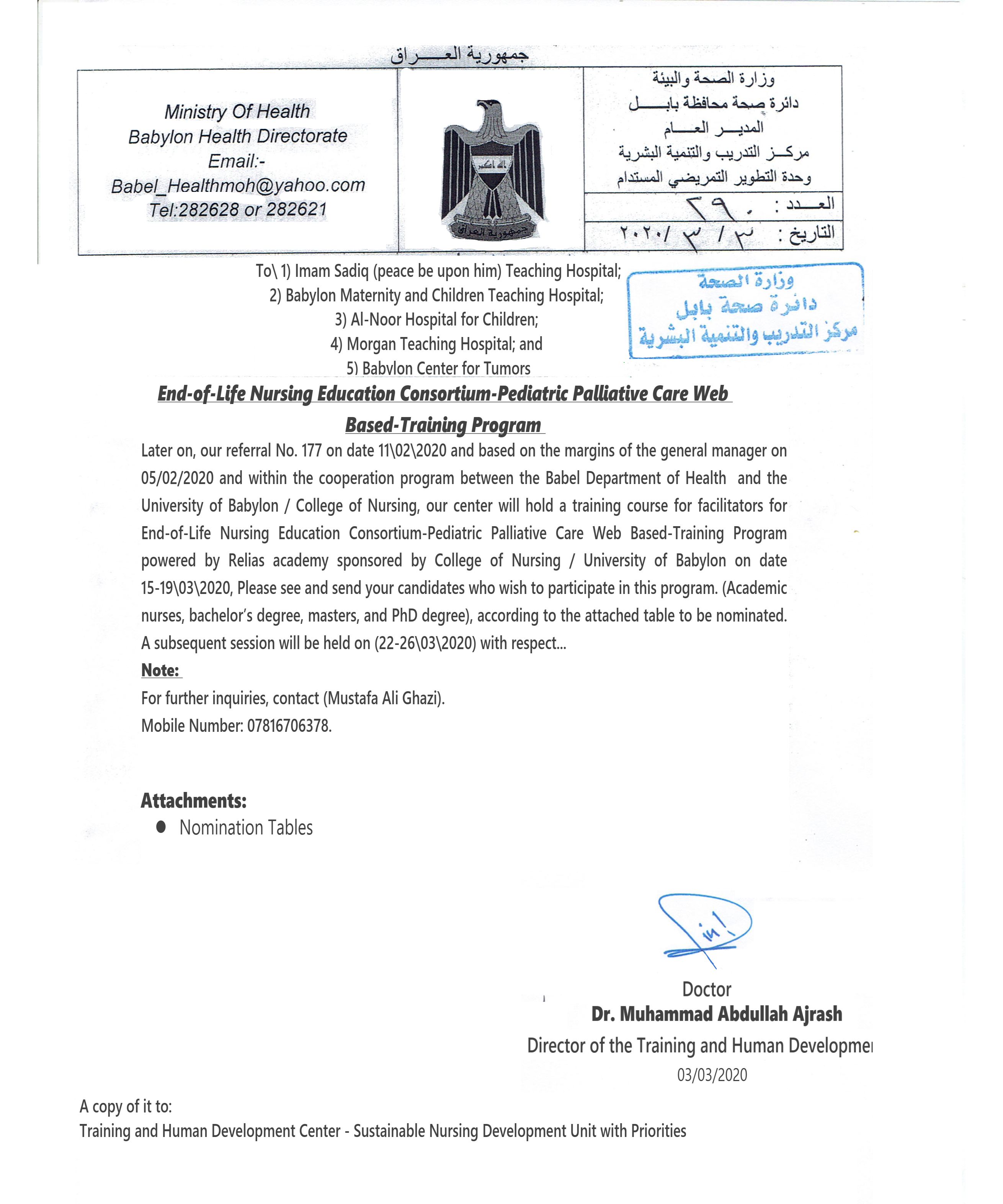

Supplement: Multimedia Appendix 3 [file resprot_v11i11e23783_app3.png]

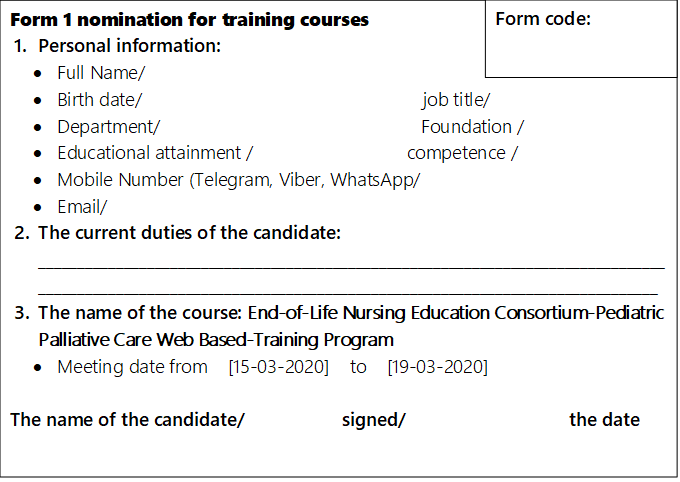

Supplement: Multimedia Appendix 4 [file resprot_v11i11e23783_app4.png]

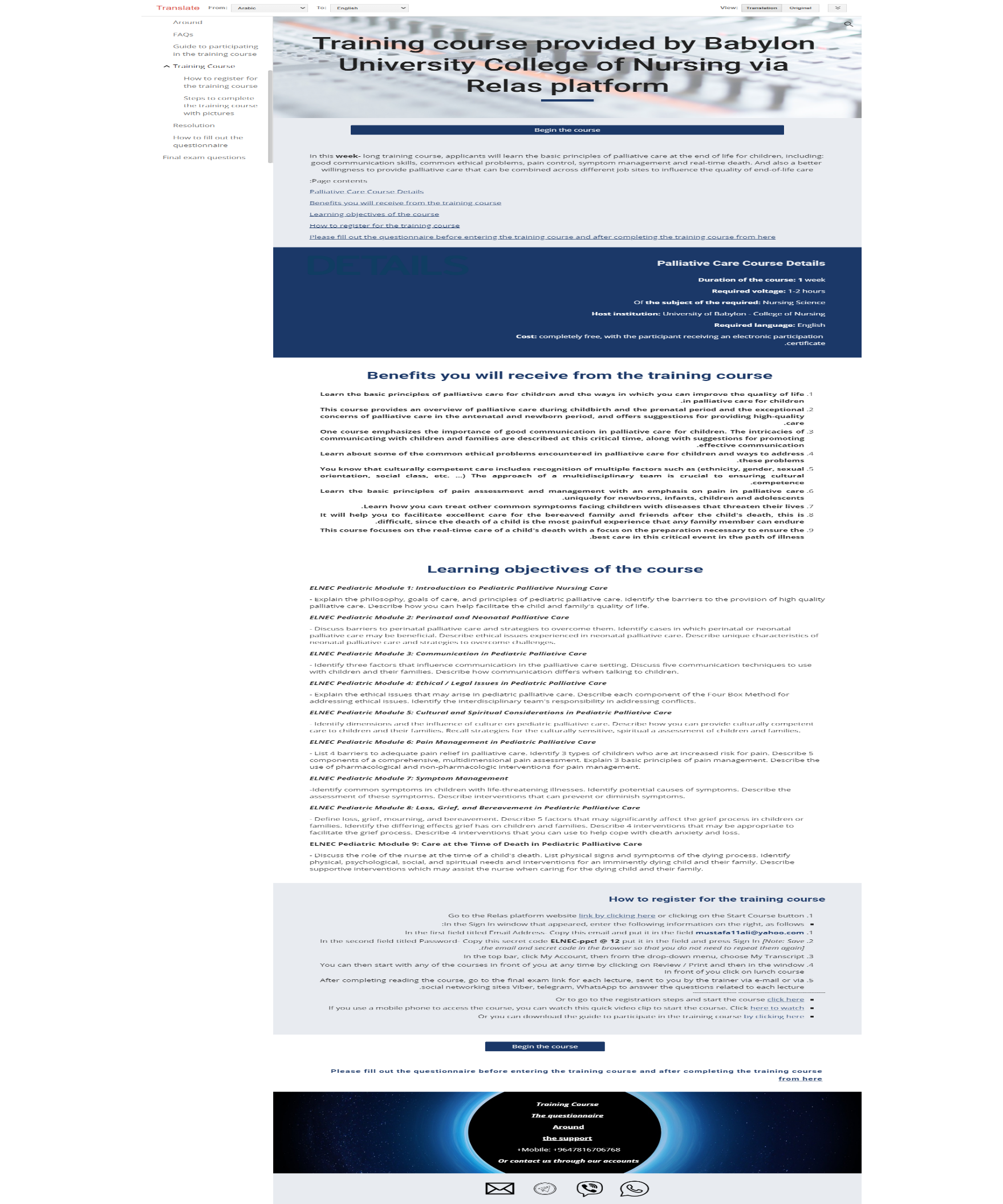

Supplement: Multimedia Appendix 7 [file resprot_v11i11e23783_app7.png]

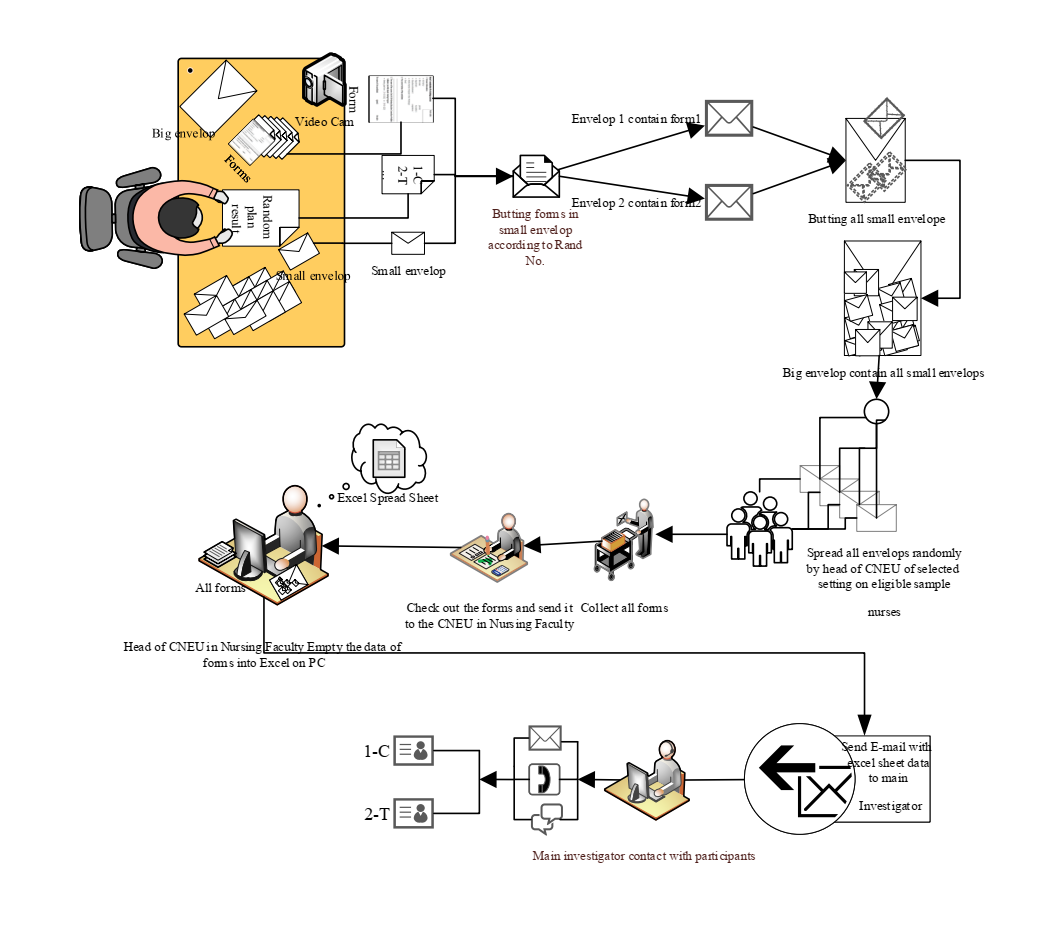

Supplement: Multimedia Appendix 8 [file resprot_v11i11e23783_app8.png]
