# Supplementary material for: Using Normalization Process Theory to Evaluate an End-of-Life Pediatric Palliative Care Web-Based Training Program for Nurses: Protocol for a Randomized Controlled Trial
Source: JMIR Res Protoc. 2022 Nov 11;11(11):e23783. doi: 10.2196/23783 (PMC9700242; doi:10.2196/23783)
Supplement: Multimedia Appendix 6 [file resprot_v11i11e23783_app6.pdf]

Form: 3<br>Date 2019/12/01

Ministry of Higher Education and Scientific Research

Faculty of Nursing - University of Babylon
Committee on Scientific Research Ethics
People's consent form to participate in scientific research
Form No. (3)

Mr. /Mrs…………………………………………..

You are invited to participate in a scientific research project entitled:
Using Normalization Process Theory to Evaluate Providing Pediatric Palliative Care at End-of-Life as Web-Based Training Intervention for Nurses: A Randomized Controlled Trial

Please take a time to read the following information carefully before deciding whether or not to participate. You can request further clarification or additional information about any matter mentioned in the form or study from the researcher or any other specialist.

**First: Search information**	
The name of the researcher	Mustafa Ali Ghazi
Supervisor's name	Prof. Amin Ajeel Yasser; A. Prof. Nuhad Mohammed Qassim
The goals	Primary Objectives-: Holding a short course ELNEC-PPC WBT program at selected Hillah city hospitals, Iraq by July2020- ; Evaluating the impact and effectiveness of this project through using NPT theory, at beginning of WBT course, after end of WBT course 2 week, , and finally at 3 months for both groups survey, outlining activities related to palliative care advocacy, applying the principles found in ELNEC-PPC WBT program for their work at selected Hillah city hospitals, Iraq by August, 2020 .Secondary Objective: -Monitoring participants for 3-months post-course in an effort to increase their Pediatric palliative care self-efficacy levels and attitudes at selected Hillah city hospitals, Iraq by August, 2020.
Expected period of a person's participation in the research	4 months (1 month for the course training and 3 months of research)
Procedures for collecting samples	1. Get approval from (Scientific Research Ethics Committee) at Babylon University / Faculty of Nursing .2. Obtain the approval of the Babil Provincial Health Department to complete the research.3 The filing of the nomination forms for the training course. 4. Contact the participant by email or mobile phone number.5. Before starting, fill out a questionnaire for the first time, then 2 weeks after completing the course, and then after 3

	months.. The researcher monitors participants for 3 months and collects data in a questionnaire prepared for this purpose.
Risks expected as a result of participating in the research	There's no risk.
Benefits to a person in exchange for participating in the research	In this two-week training course, applicants will learn the basic principles of end-of-life palliative care for children, including: new communication skills and problems. Common moral, pain control, symptom management and real-time care of death.

**Second: Information for the person involved in the search**
1. Participation in this research is voluntary
2. You can pull your participation from studying whenever you want and for whatever reason
3. You have the right not to answer any question you do not want to answer
4. Your participation in the research will not incur any financial expenses.
5. Your participation in the research does not result in any accountability that may harm you personally or your work.
6. Your name will be confidential and the information resulting from your participation will be treated with complete confidentiality and will not be shared with anyone except the researcher, supervisor and ethics committee if necessary.
7. The information you have provided and the scientific results of this research are for scientific purposes only and there will be no reference to you or your family in any publication of this study.
8. You have the right to know the general results of the research, or any results that are specific to you.

Third: Contact information
If there is any question or complaint from you about the research project, you can contact the researcher or the Research Ethics Committee at Babylon University - Faculty of Nursing
The name of researcher Mustafa Ali Ghazi phone number 07816706378 email **mostafa.ghazi@uobabylon.edu.iq**

Form: 3<br>Date 2019/12/01

Ministry of Higher Education and Scientific Research

Faculty of Nursing - University of Babylon
Committee on Scientific Research Ethics
People's consent form to participate in scientific research

Form No. (3)

Committee of Ethics of Scientific Research - University of Babylon - Faculty of Nursing: Tel. 00964772850116 Email ammar_shalan@yahoo.com.au

Participant name:<br><br><br><br>Signed:<br>Date:	If the person is under the age of 18 years of age or is unable to understand or read the form, please sign his legal guardian.<br>The name of the joint guardian:<br><br>Kinship:<br><br>Signed:<br><br>Date:
