# Supplementary material for: Using Normalization Process Theory to Evaluate an End-of-Life Pediatric Palliative Care Web-Based Training Program for Nurses: Protocol for a Randomized Controlled Trial
Source: JMIR Res Protoc. 2022 Nov 11;11(11):e23783. doi: 10.2196/23783 (PMC9700242; doi:10.2196/23783)
Supplement: Multimedia Appendix 9 [file resprot_v11i11e23783_app9.pdf]

Ministry of Higher Education
& Scientific Research
University of Babylon
College of Nursing

NoMAD Tool

Survey Instructions

This survey is designed to help get a better understanding of how to apply and integrate new technologies and complex interventions in health care.

This survey asks questions about the implementation of **End-of-Life Nursing Education Consortium- pediatric Palliative Care Web Based-Training Program (ELNEC- PPC WBT) program in provision of pediatric palliative care at end of life**. We understand that people involved with **pediatric palliative care at end of life** have different roles, and that people may have more than one role.

For this survey, please answer all the statements from the perspective of this role. Depending on your role or responsibilities in pediatric palliative care at end of life, some statements may be more relevant than others.

The survey is in 3 parts. Part A asks some brief questions about yourself and your role. Part B includes three general questions about **pediatric palliative care at end of life.** Part C contains a set of more detailed questions about **pediatric palliative care at end of life** for each statement in Part C, there is the option to agree or disagree with what is being asked **(OPTION A).** However, if you feel that the statement is not relevant to you, there are also options to tell us why **(OPTION B).**

Please take the time to decide which answer **best suits your experience for each statement and tick the appropriate circle**

Part A: About yourself

1. Gender:

○ Female ♀ ○ Male ⚦

2. Age

○ Young Adulthood 20–40 years ○ Middle Adulthood 40–65 years

3. How many years have you worked for this hospital unit? ***(If your Trust has merged with another or changed its name, please include in your answer all the time you have worked with this Trust and its predecessors)***

○ Less than one year ○ 1-2 years ○ 3-5 years ○ 6-10 years ○ 11-15 years ○ More than 15 years

4. How would you describe <u>your professional job category</u>?

○ Intensive Care Unit Nurse ○ Medical-Surgical Nurse ○ Emergency Room Nurse ○ Operating Room Nurse ○ Post- Anaesthesia Care Unit Nurse ○ Nurse Manger

○ Staff Nurse ○ Pediatric Nurse ○ Pediatric Intensive Care Unit Nurse ○ Labour and Delivery Nurse ○ Cardiovascular Nurse ○ Oncology Nurse

○ Other____________

5. Academic Qualification

○ Bachelor's Graduate ○ High Diploma Graduate ○ Master Postgraduate ○ Doctoral Postgraduate

6. What is your current job title (choose a job address)

○ Academic Nurse ○ Specialist Nurse ○ Other____________

7. The number of local training courses that participated for the computer:

○ I Did not get any session ○ One-session ○ Two sessions ○ 3 or more sessions

8. The number of local training courses that participated for pediatric palliative care:

○ I Did not get any session ○ One-session ○ Two sessions ○ 3 or more sessions

Part B: General questions about the pediatric palliative care at end of life

When you use pediatric palliative care at end of life, how familiar does it feel?

Still feels very new **Feels completely familiar**

0 1 2 3 4 5 6 7 8 9 10

Do you feel the pediatric palliative care at end of life is currently a normal part of your work?

Not at all **Somewhat** **Completely**

0 1 2 3 4 5 6 7 8 9 10

Do you feel pediatric palliative care at end of life will become a normal part of your work?

Not at all **Somewhat** **Completely**

0 1 2 3 4 5 6 7 8 9 10

Part C: Detailed questions about the pediatric palliative care at end of life

For each statement please select an answer that best suits your experience using Option A. If the statement is not relevant to you please select an answer from Option B.

Section C1		Option A: Strongly Agree	Agree	Neither agree nor disagree	Disagree	Strongly disagree	Option B: Not relevant to my role	Not relevant at this stage	Not relevant to the intervention
1.	I can see how the pediatric palliative care at end of life differs from usual ways of working	○	○	○	○	○	○	○	○
2.	Staff in this organisation have a shared understanding of the purpose of the pediatric palliative care at end of life	○	○	○	○	○	○	○	○
3.	I understand how the pediatric palliative care at end of life affects the nature of my own work	○	○	○	○	○	○	○	○
4.	I can see the potential value of the pediatric palliative care at end of life for my work	○	○	○	○	○	○	○	○

For each statement please select an answer that best suits your experience using Option A. If the statement is not relevant to you please select an answer from Option B.

Section C2	Option A: Strongly Agree	Option A: Agree	Option A: Neither agree nor disagree	Option A: Disagree	Option A: Strongly disagree	Option B: Not relevant to my role	Option B: Not relevant at this stage	Option B: Not relevant to the intervention
1. There are key people who drive the pediatric palliative care at end of life forward and get others involved	○	○	○	○	○	○	○	○
2. I believe that participating in the pediatric palliative care at end of life is a legitimate part of my role	○	○	○	○	○	○	○	○
3. I'm open to working with colleagues in new ways to use the pediatric palliative care at end of life	○	○	○	○	○	○	○	○
4. I will continue to support the pediatric palliative care at end of life	○	○	○	○	○	○	○	○

For each statement please select an answer that best suits your experience using Option A. If the statement is not relevant to you please select an answer from Option B.

Section C3		Option A					Option B		
		Strongly Agree	Agree	Neither agree nor disagree	Disagree	Strongly disagree	Not relevant to my role	Not relevant at this stage	Not relevant to the intervention
1.	I can easily integrate the pediatric palliative care at end of life into my existing work	○	○	○	○	○	○	○	○
2.	the pediatric palliative care at end of life disrupts working relationships	○	○	○	○	○	○	○	○
3.	I have confidence in other people's ability to use the pediatric palliative care at end of life	○	○	○	○	○	○	○	○
4.	Work is assigned to those with skills appropriate to the pediatric palliative care at end of life	○	○	○	○	○	○	○	○
5.	Sufficient training is provided to enable staff to implement the pediatric palliative care at end of life	○	○	○	○	○	○	○	○
6.	Sufficient resources are available to support the pediatric palliative care at end of life	○	○	○	○	○	○	○	○
7.	Management adequately supports the pediatric palliative care at end of life	○	○	○	○	○	○	○	○

For each statement please select an answer that best suits your experience using Option A. If the statement is not relevant to you please select an answer from Option B.

Section C4		Option A					Option B		
		Strongly Agree	Agree	Neither agree nor disagree	Disagree	Strongly disagree	Not relevant to my role	Not relevant at this stage	Not relevant to the intervention
1.	I am aware of reports about the effects of the pediatric palliative care at end of life	○	○	○	○	○	○	○	○
2.	The staff agree that the pediatric palliative care at end of life is worthwhile	○	○	○	○	○	○	○	○
3.	I value the effects that the pediatric palliative care at end of life has had on my work	○	○	○	○	○	○	○	○
4.	Feedback about the pediatric palliative care at end of life can be used to improve it in the future	○	○	○	○	○	○	○	○
5.	I can modify how I work with the pediatric palliative care at end of life	○	○	○	○	○	○	○	○

SURVEY CONCLUSION

Thank you for completing our survey.
